# Supplementary figures and images for: Using machine learning to assess the extent of busy ambulances and its impact on ambulance response times: A retrospective observational study
Source: PLoS One. 2024 Jan 5;19(1):e0296308. doi: 10.1371/journal.pone.0296308 (PMC10769093; doi:10.1371/journal.pone.0296308)

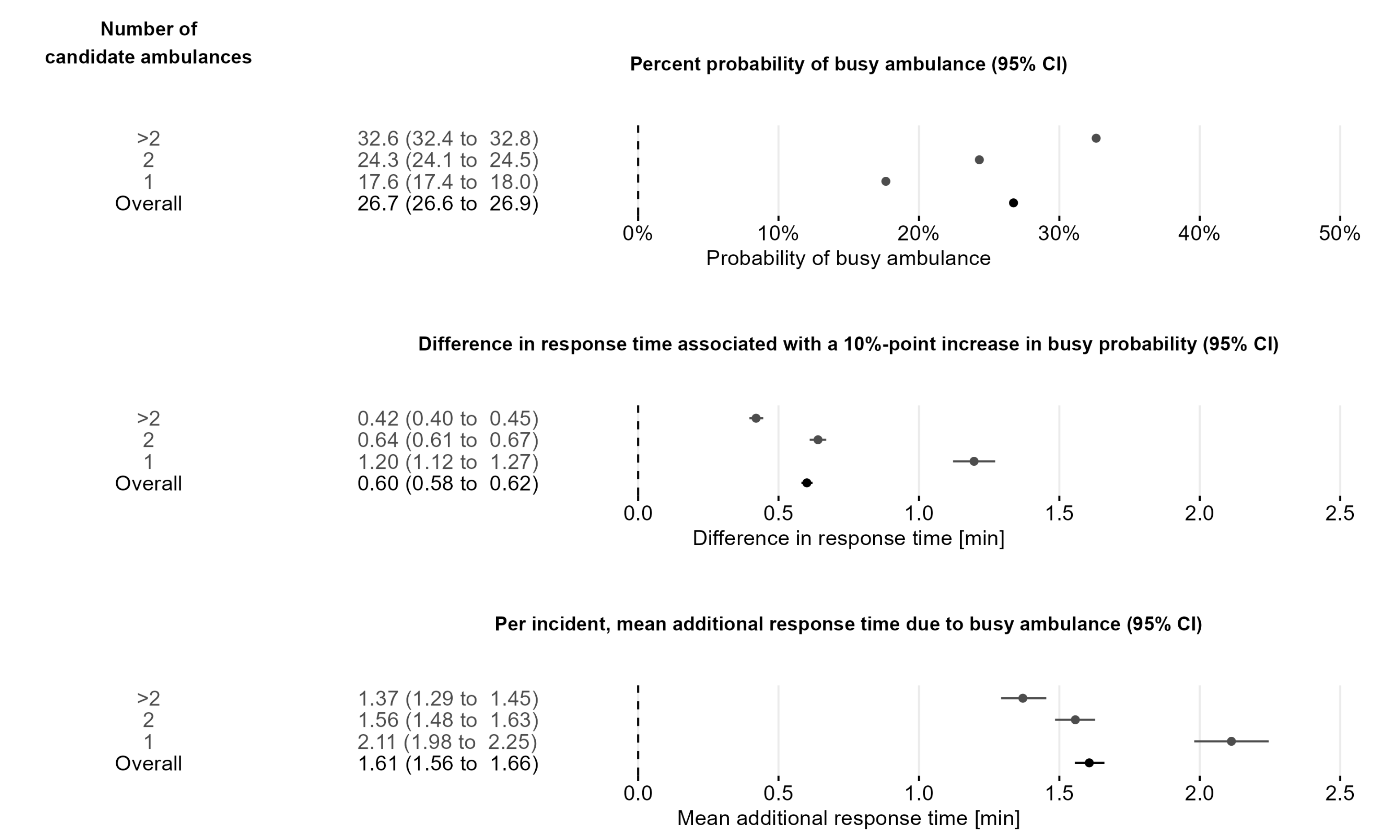

Supplement: S1 Fig — Candidate ambulance is defined as an ambulance with more than 10% probability of responding to an incident. (TIF) [file pone.0296308.s001.tif]

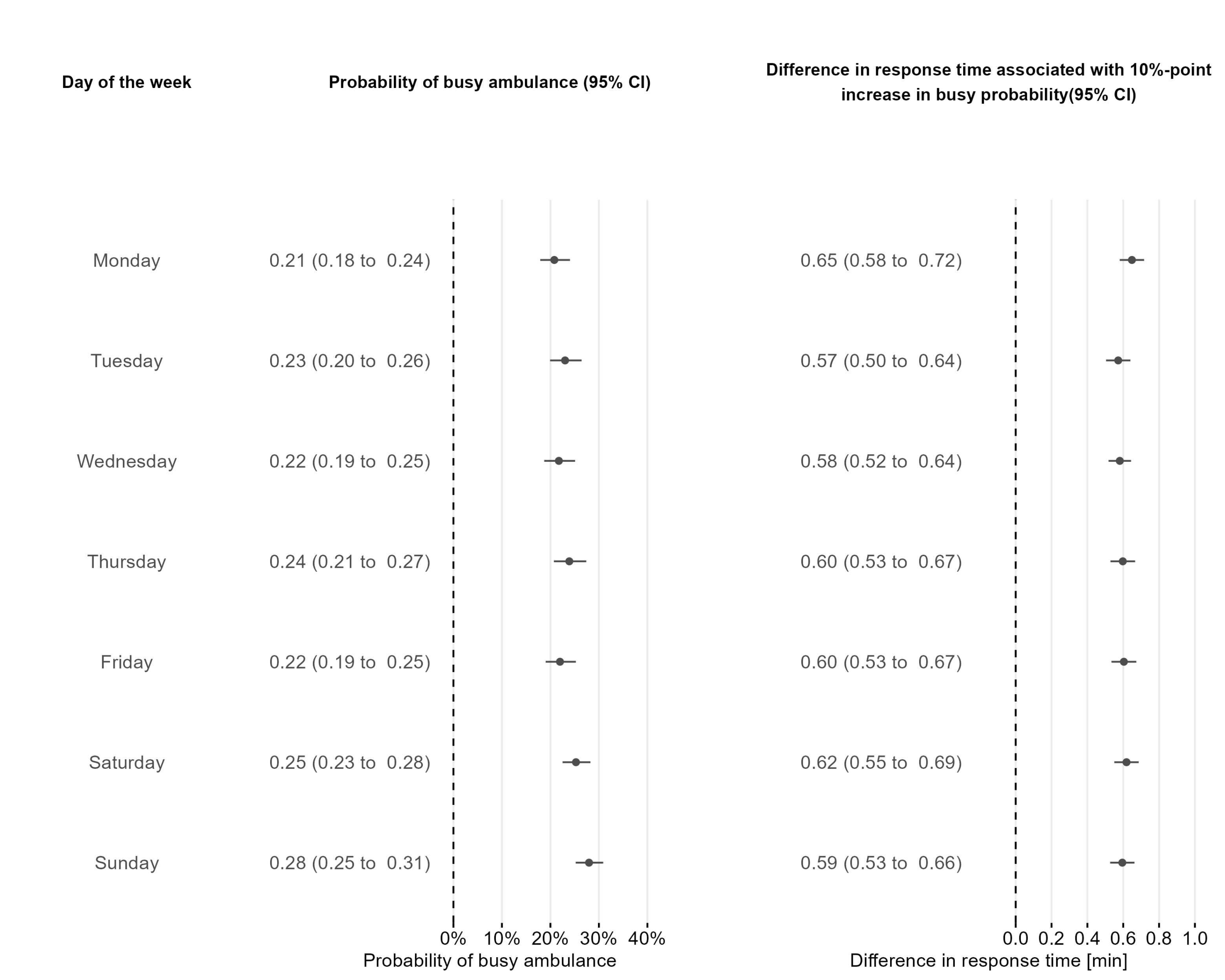

Supplement: S2 Fig — Differences in response time were computed within the neighbourhood and year and adjusted for hour of the day, day of the week, and month. (TIF) [file pone.0296308.s002.tif]

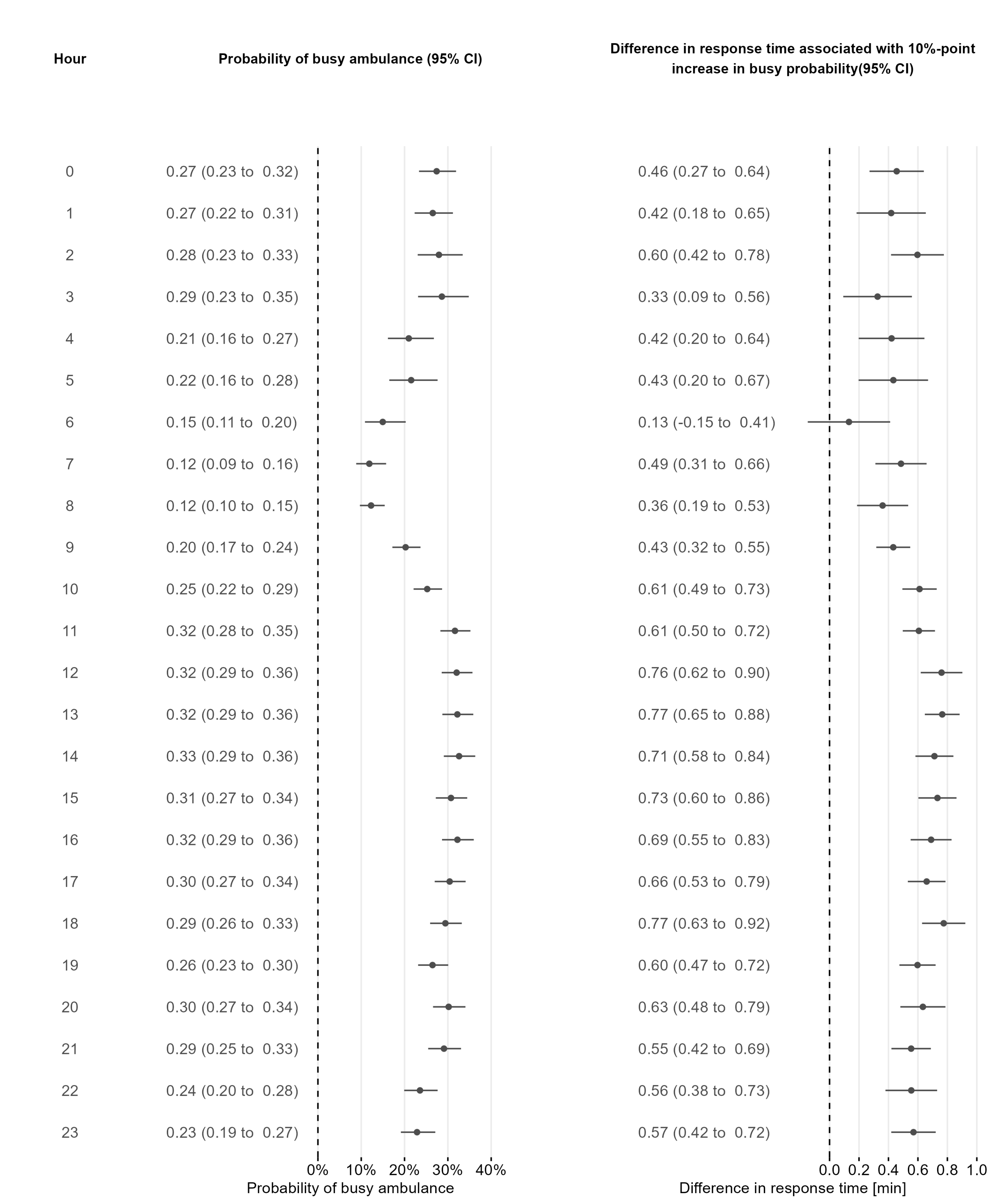

Supplement: S3 Fig — Differences in response time were computed within the neighbourhood and year and adjusted for hour of the day, day of the week, and month. (TIF) [file pone.0296308.s003.tif]

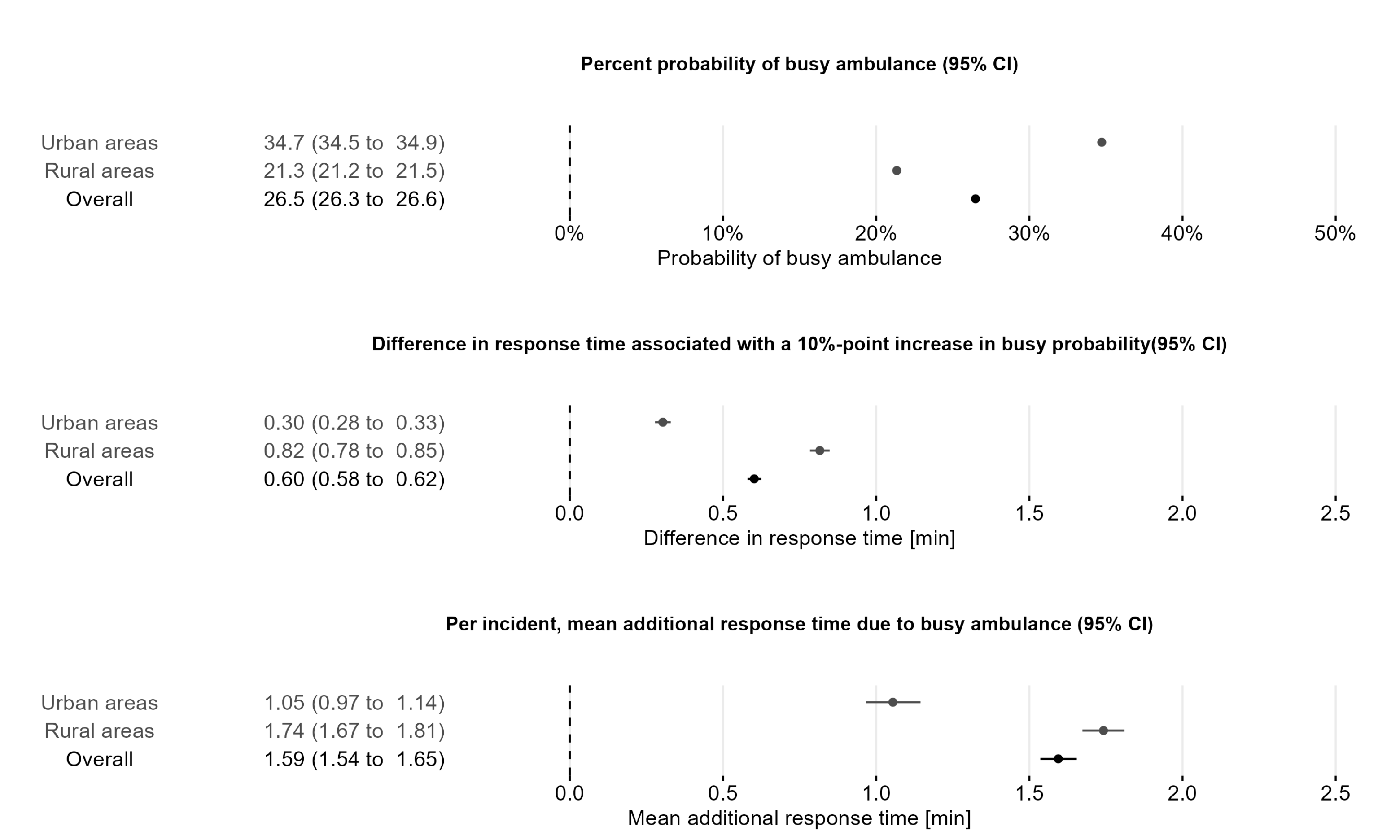

Supplement: S4 Fig — Differences in response time were computed within neighbourhood and year and adjusted for hour of the day, day of the week, and month. (TIF) [file pone.0296308.s004.tif]
