# Supplementary material for: Using machine learning to assess the extent of busy ambulances and its impact on ambulance response times: A retrospective observational study
Source: PLoS One. 2024 Jan 5;19(1):e0296308. doi: 10.1371/journal.pone.0296308 (PMC10769093; doi:10.1371/journal.pone.0296308)
Supplement: S1 Table — (DOCX) [file pone.0296308.s005.docx]

**S1 Table. Odds ratio for the incident being one of the six most common incident types associated with a 10% increase in busy probability computed within the neighbourhood and year and adjusted for hour of the day, day of the week, and month.**

| **Type of incident** | **Number of incidents** | **Odds ratio** | **95% confidence interval** |
| --- | --- | --- | --- |
| Acute illness | 148,581 | 0.990 | 0.986 to 0.994 |
| Accident | 15,278 | 1.005 | 0.998 to 1.012 |
| Transport to hospital | 10,346 | 1.006 | 0.997 to 1.015 |
| Traffic accident | 9,900 | 1.000 | 0.991 to 1.010 |
| Psychiatry or intoxication | 7,191 | 0.998 | 0.988 to 1.008 |
| Fire | 3,355 | 0.994 | 0.979 to 1.009 |
