# Supplementary material for: Using machine learning to assess the extent of busy ambulances and its impact on ambulance response times: A retrospective observational study
Source: PLoS One. 2024 Jan 5;19(1):e0296308. doi: 10.1371/journal.pone.0296308 (PMC10769093; doi:10.1371/journal.pone.0296308)
Supplement: Attachment — Submitted filename: Busy ambulances_Response to Reviewers_PO.docx [file pone.0296308.s006.docx]

Response to Reviewers

**Using machine learning to assess the extent of busy ambulances and its impact on ambulance response times: A retrospective observational study**

**PONE-D-23-26575**

Lars Eide Næss, Andreas Jørstad Krüger, Oddvar Uleberg, Helge Haugland, Jostein Dale, Jon-Ola Wattø, Sara Marie Nilsen, Andreas Asheim

**Dear Editor,**

**Dear Reviewers,**

The authors would like to thank the Editor and Reviewers for their valuable and constructive feedback on our article. We highly appreciate your suggestions in helping to improve the presentation of our study results and giving us the opportunity to correct the manuscript.

In the following is our point-by-point response to your comments and suggestions. We sincerely hope that this addresses all your concerns regarding the manuscript.

In our re-submission you will find a “track changes”-version (marked TC) of the revised manuscript with yellow markings for all changes. The revised manuscript has been sent to American Journal Experts (AJE.com) for re-editing before submission. We have also updated all figures to new versions, correcting a minor spelling error and adjusting resolution. Minor changes in results reflects small variations due to new computations.

Trondheim, 9 November 2023

*Lars E. Næss, on behalf of the authors*

# Editor comments (#E)

### #E-1

*Please ensure that your manuscript meets PLOS ONE's style requirements, including those for file naming. The PLOS ONE style templates can be found at* 
[*https://journals.plos.org/plosone/s/file?id=wjVg/PLOSOne_formatting_sample_main_body.pdf*](https://eur03.safelinks.protection.outlook.com/?url=https%3A%2F%2Fjournals.plos.org%2Fplosone%2Fs%2Ffile%3Fid%3DwjVg%2FPLOSOne_formatting_sample_main_body.pdf&data=05%7C01%7CAndreas.Asheim%40stolav.no%7C383d8fc4edb0486d24d808dbc4a6dd63%7C92c8809f91e0445b804fb6a7b43ef73a%7C0%7C0%7C638319993317891086%7CUnknown%7CTWFpbGZsb3d8eyJWIjoiMC4wLjAwMDAiLCJQIjoiV2luMzIiLCJBTiI6Ik1haWwiLCJXVCI6Mn0%3D%7C3000%7C%7C%7C&sdata=ifLlVZkTLdkT%2FNBe0sCx%2FV%2Ft4uR1gN3kYdYM69r4NOw%3D&reserved=0) *and* 
[*https://journals.plos.org/plosone/s/file?id=ba62/PLOSOne_formatting_sample_title_authors_affiliations.pdf*](https://eur03.safelinks.protection.outlook.com/?url=https%3A%2F%2Fjournals.plos.org%2Fplosone%2Fs%2Ffile%3Fid%3Dba62%2FPLOSOne_formatting_sample_title_authors_affiliations.pdf&data=05%7C01%7CAndreas.Asheim%40stolav.no%7C383d8fc4edb0486d24d808dbc4a6dd63%7C92c8809f91e0445b804fb6a7b43ef73a%7C0%7C0%7C638319993317891086%7CUnknown%7CTWFpbGZsb3d8eyJWIjoiMC4wLjAwMDAiLCJQIjoiV2luMzIiLCJBTiI6Ik1haWwiLCJXVCI6Mn0%3D%7C3000%7C%7C%7C&sdata=XhyDc%2B0x4GvN6S1FLPrciBEF87dc927TQrDiXBKaZUY%3D&reserved=0)

**Author’s reply**

We have revised the manuscript and supplementary materials, hopefully addressing all your style requirement issues.

### #E-2

*We note that you have indicated that data from this study are available upon request. PLOS only allows data to be available upon request if there are legal or ethical restrictions on sharing data publicly. For more information on unacceptable data access restrictions, please see* [*http://journals.plos.org/plosone/s/data-availability#loc-unacceptable-data-access-restrictions*](http://journals.plos.org/plosone/s/data-availability#loc-unacceptable-data-access-restrictions)*.*

*In your revised cover letter, please address the following prompts:*

*a) If there are ethical or legal restrictions on sharing a de-identified data set, please explain them in detail (e.g., data contain potentially sensitive information, data are owned by a third-party organization, etc.) and who has imposed them (e.g., an ethics committee). Please also provide contact information for a data access committee, ethics committee, or other institutional body to which data requests may be sent.*

*We will update your Data Availability statement on your behalf to reflect the information you provide.*

**Author’s reply**

We do not have permission to share any underlying data, as they are restricted by legal and ethical regulation. The use of location and time stamps, in combination with the use of ambulance, or other health resources, could be used to identify incidents or patients. Permission to access data could be granted through a formal request to the regional health authority, Central Norway Regional Health Authority, P.o.box 464, N-7501 Stjørdal, Norway (https://www.helse-midt.no/). We have chosen to publish code for our analysis on GitHub^[1]^, so that our method could be validated using the same data or similar data from other emergency medical communication centre and ambulance services.

We have updated the cover letter. To address this point for readers, we have also added a data-sharing statement in our manuscript (*line #142*).

### #E-3

*We note that [Figure 1] in your submission contain [map/satellite] images which may be copyrighted. All PLOS content is published under the Creative Commons Attribution License (CC BY 4.0), which means that the manuscript, images, and Supporting Information files will be freely available online, and any third party is permitted to access, download, copy, distribute, and use these materials in any way, even commercially, with proper attribution. For these reasons, we cannot publish previously copyrighted maps or satellite images created using proprietary data, such as Google software (Google Maps, Street View, and Earth). For more information, see our copyright guidelines:* [*http://journals.plos.org/plosone/s/licenses-and-copyright*](http://journals.plos.org/plosone/s/licenses-and-copyright)*.*

*We require you to either (1) present written permission from the copyright holder to publish these figures specifically under the CC BY 4.0 license, or (2) remove the figures from your submission:*

**Author’s reply**

We have been in contact with the Norwegian ArcGis distributor, Geodata AS, and obtained the necessary CC BY 4.0 permission for printing the figure. Please see uploaded “Request for permission to Publish content under CC” in the editorial manager. The figure legend has also been changed according to requirements with a designated CC BY 4.0 marking and specified to cover both main and overview maps *(line #106).*

### #E-4

*We notice that your supplementary [figures/tables] are included in the manuscript file. Please remove them and upload them with the file type 'Supporting Information'. Please ensure that each Supporting Information file has a legend listed in the manuscript after the references list.*

**Author’s reply**

S1 Table is now removed from the manuscript and uploaded as a separate file. Supporting information with corresponding legends at the end of the manuscript has been updated accordingly.

#E-5, Additional Editor Comments
*The manuscript has been reviewed by two referees. Their recommendations are consistent. Overall, they believe that the work has the potential to be published but there are still a number of clarification issues need to be addressed. I recommend a Major Revision for the authors to address their concerns.*

**Author’s reply**

We thank you for your positive and constructive feedback. We have followed up on your comments to improve our manuscript and hope it is eligible for publication.

# Reviewer 1’s comments (#R1)

## #R1-INTRO

*I reviewed the article by Lars Eide Næss et al, entitled “Extent of busy ambulances and the impact on ambulance response times: A cohort study” submitted to PLOS ONE (Manuscript ID: PONE-D-23-26575). In this retrospective observational study, the authors mainly assessed the relationship between prevalence of busy ambulances and differences in response times. The authors found that busy ambulances are associated with increased response times, and this effect was particularly evident in rural area. First, the reviewer respects for the Authors' tremendous effort and time spent on this manuscript. However, there are several concerns regarding this manuscript, which are listed below:*

### #R1-1

*This reviewer feels that the retrospective observational study is more suitable term for describing this study. Please consider to replace cohort study with retrospective observational study, thought the manuscript.*

**Author’s reply**

We agree and have changed our manuscript accordingly. This is also reflected in the revised manuscript title *(line #1).*

## METHODOLOGY

### #R1-2

*The authors stated that "We used machine learning techniques on data from nearby incidents to estimate the weighted probability of up to five different ambulances responding to medical emergencies". This reviewer, as well as many readers are not so familiar with machine learning techniques. Please describe more in details used technique, with relevant references.*

**Author’s reply**

The description of the Methods was not sufficiently detailed. We have revised this section, hopefully to the better *(line #153).*

### #R1-3

*Who planned this study, who collected data, and who conducted the statistical analysis? I think if the same researchers are involved in study planning, data collecting, outcome measurement, and statistical analysis, there is a theoretical risk of biased assessment.*

**Author’s reply**

The study was planned in cooperation with all the authors, spanning a wide range of competence from different parts of the EMS service in Central Norway (service managers, physicians and researchers). All data and analytical work was done directly on service data from the regional health authority data warehouse, which included data on all medical emergency incidents in the region. The handling of data was mainly done by Andreas Asheim together with Lars E. Næss. Author contributions are listed in the submission system according to the CRediT taxonomy.

We cannot rule out bias in our assessment. However, we did take steps to avoid this, for example by working out the steps in the analysis before examining results. We used objective analysis criteria, and sincerely feel that our assessment was unbiased with regards to both analysis and outcome. To clarify this point, we have added a paragraph in the Strengths and limitations section *(line #350).*

### #R1-4

*Describe any efforts to address potential sources of bias. For example, blinded exposure is one attractive method to address this issue.*

**Author’s reply**

We performed all work on already collected observational real world data from the service’s information system, which comprises all medical emergency incidents in the region. There is thus no risk of bias from selection of events. Bias from so-called researcher degrees of freedom, were addressed by running analyses according to a pre-specified protocol^[2]^.

The main risk of bias in such studies are surely from indication, i.e., that sicker patients get a quicker response, such that geographical and temporal variability in indications may confound the analysis. Notably, patient indications are not feasible to account for directly, since all factors that enter into the assessment of the operator are not available in data. These are confounders that can’t be measured, regardless of the amount of data we analyse. Our strategy to handle this unmeasured confounding was based on a natural experiment: When considering incidents at the same location at similar times, the availability of ambulances are likely not associated with patient indications, in which case indication bias is minimised. Note that the balance test support this claim *(S1 Table).*

This point should have been made clearer, therefore we added more details in the Statistics section on the strategy for addressing sources of bias *(line #197)*.

### #R1-5

*Sample size calculation is missing. Explain how the study size was arrived at. If sample size was not determined a priori, please state so and provide post-hoc sample size estimation to provide the estimation of how was the power of this study.*

**Author’s reply**

This was an observational study based on a complete set of data from ten years of ambulance missions in our region. Prior data was available, but not of sufficient quality *(cf. query #R1-9).* We had thus no influence on the study size. We prefer not to provide post-hoc sample size calculations, since such calculations are prone to overestimate power and are not advised according to Gelman^[3]^ and Zhang et al.^[4]^.

### #R1-6

*Explain how missing data were addressed.*

**Author’s reply**

As described in *“Data and study cohort”* and *Fig 2*. *(flowchart),* we excluded ambulance missions registered in the EMCC database with missing coordinates or “bad time stamps”, defined as negative response time or response time bigger than 6 hours. In total 16% (32,505) of the registered incidents had such shortcomings. We don’t know for certain why some missions are lacking these parameters, but many of these cases are likely aborted missions, malfunctions in equipment and random user errors. Given the analysis design, we assume that excluding incidents with missing data will lead to a non-differential error, not biasing the results. This assumption is hard to test, and bias cannot be ruled out, of course. This point has now been added in the Strengths and limitations section (line #337).

### #R1-7

*The rationale of the definition of busy ambulance should be described greater in details. Were there any supporting references?*

**Author’s reply**

We agree that the definition of busy ambulance needs a better description in our manuscript, and this section has been expanded (*line #153*). We added a reference (*ref. #22*) to point readers to the multinomial regression. However, our way of assessing busy ambulances is novel *(line #276).*

### #R1-8

*Clearly define all potential confounders, and effect modifiers in method section. This study does not consider the any confounders.*

**Author’s reply**

We have elaborated on this in the Statistics section in our revised manuscript (*line #197*), see also reply to comment *#R1-4.*

### #R1-9

*Please give the characteristic of the data source. How did you do to assure the quality of data? Since this is a retrospective observational study, quality assurance is of vitally important.*

**Author’s reply**

The data are real world data from our local EMCC’s from the past ten years. In this period, data were rigorously registered by the EMCC operator and ambulance personnel for each incident, and much of the data are registered automatically. Therefore we have much confidence in this data. Note that the conclusions of the paper are based on time stamps and coordinates, whereas other less reliable variables are not relied upon. This has now been further emphasised in the Strengths and limitations section (*line #329*). As described in the answer to comment *#R1-6*, there are some incomplete data, but we have no reason to believe these result in faulty conclusions.

## RESULTS

### #R1-10

*Table1: Can the authors provide the patient characteristics (age, year, etc), vital sings, aetiology (trauma, cerebral infarction, acute myocardial infarction, etc), and degree of urgency? Such essential data is missing. At this current form, many readers including myself find it difficult to image the characteristics of study subjects. There are too many unmeasured confounders, which hiders the meaningful comparison.*

**Author’s reply**

We did not have access to any patient data for this study, as our goal was to look at the relationship between busy ambulances and ambulance response times solely *(line #149, #282, #338 and #360*). From the EMCC database we did have access to level of urgency and type of mission. Regarding unmeasured confounders, we argue that our method was designed to handle this, as discussed above *(#R1-8, line #197*). Also note that the additional analyses support that there were no substantial association between busy ambulances and types of mission (*S1 Table*).

### #R1-11

*COVID 19 pandemic can be the significant confounders. The authors should assess the effect of COVID19 pandemic or some other confounders on measured outcomes.*

**Author’s reply**

We agree that Covid-19 likely had an impact both on incident prevalence, incident profile and ambulance response. To assess the impact of this on our results, we included a separate analysis excluding the years 2020 and 2021 *(S4 Fig).* This did not substantially change the results (*line #222 and #271*).

### #R1-12

*The authors state that ambulance response time may impact morbidity and mortality, especially among patients with time-critical conditions. Can author provide the data regarding association among prevalence of busy ambulances, increased response time and patients outcome (e.g. survival rate) in these time sensitive population?*

**Author’s reply**

As described under comment *#R1-10*, we did not have any data on patient outcomes (*line #149*). This is future work, a follow up paper is being planned *(line #360).*

### #R1-13

*Are observed association between prevalence of busy ambulances and differences in response times are clinically meaningful? What is your opinion on this?*

**Author’s reply**

This is a good question. As we did not have access to any patient data we could only speculate in the clinical relevance, but, as mentioned under comment #R1-12, this will be further addressed in future work (line #360). We have tried to elaborate more on the relevance of our findings in an organisational setting in the revised Discussion.

## DISCUSSION

### #R1-14

*Limitation section needs substantial revision. Please consider the important limitations and do not just list them but consider their relevance and how they might bias the results. Discuss both direction and magnitude of any potential bias.*

**Author’s reply**

We have revised our Strengths and limitations section to address your concerns about relevance and bias in detail (*line #340*).

### #R1-15

*Discuss the generalizability (external validity) and Implications for practice of the study results. Give a cautious overall interpretation of results considering objectives, limitations, multiplicity of analyses, results from similar studies, and other relevant evidence.*

**Author’s reply**

The connection between busy ambulances and prolonged ambulance response times are intuitive, but we wanted to find a method for assessing the extent of this in real world data. Our results show that there is a strong connection between busy ambulances and response times in a wide variety of settings (e.g. urban/rural, seasons/day of week/time of day, and with different levels of vulnerability (available resources)). Our result, in terms of magnitude of effect, is specific for our regional context, but we believe that our underlying findings are generalisable to most services and would encourage others to test our method. We have addressed external validity in our revised Discussion (*line #324*).

### #R1-16

*Please indicate future research direction more in details, immediately after limitation section.*

**Author’s reply**

We have added more info about future research, in accordance with what is mentioned in comment #*R1-12 (line #360).*

## CONCLUSION

### #R1-17

*The authors concluded that "Using machine learning on observational data to calculate an indicator for busy ambulances showed potential for further research on ambulance response times and patient outcomes" The reviewer thinks this is not objective.*

**Author’s reply**

We agree, our conclusion has been revised (*line #364*).

### #R1-18

*The authors should provide the minimal anonymized data set used in this manuscript, according to the journal's policy.*

**Author’s reply**

As stated under the answer to editor’s comment *#E2*, we are not allowed to share any data due to legal and ethical regulations (*line #142*). It is possible to apply to the regional health authority for data access. We have shared the relevant code^[5]^ to recreate our study on similar data, and would be happy to assist if needed.

## #R1-SUMMARY

*Although the number of criticisms listed above, this reviewer should however state that it is laudable that this work is derived from huge efforts made by the authors, who are working as the frontline healthcare professionals. The reviewer respects the authors’ time and effort spent on this manuscript, and the authors ‘patience and professionalism in dealing with my comments.*

**Author’s reply**

We would like to take the reviewer for their contribution in evaluating our manuscript and giving valuable feedback. We hope that our revised manuscript can bring clarity and answer all your concerns.

# Reviewer 2’s comments (#R2)

## #R2-INTRO

*Thank you for this important subject and this nice paper. However, I believe it could be improved and presented more reproducibly to the audience. Here are some suggestions:*

### #R2-1

*You mentioned in the methods, “If there is an acute need for an ambulance, the EMCC operator sends the most suitable available resource to the incident, often a road ambulance from the closest ambulance station. Nevertheless, the services operate in a dynamic setting where the operator’s decision may depend on circumstances. Several ambulances may service the same area, especially in densely populated areas, and ambulances on transport missions may be rerouted to attend acute incidents.” I wonder if it is possible to provide numbers about this time that the operator can take to find the most appropriate unit and its variation and to explain in the method what you did do to account for such confounding factor, specifically here time from receiving an emergency call and finding the most appropriate unit. Also, how much time it takes a unit to move upon receiving the call?*

**Author’s reply**

Thanks’ for an interesting question. First we would like to address your concern about EMCC coordination as a confounding factors. In incidents where the most obvious resource is unavailable, the EMCC operator must assess another solution. The EMCC operator has a consecutive overview over the local ambulance fleet, and are free to dispose any available unit. This helps the operator to make a quick decision, and we therefore believe the subsequent delay is marginal. However, there may be some delay in the dispatch from the EMCC in case of busy ambulances. This is a part of the total response time, as defined. Note that the causal arrow goes from busy ambulances via delayed dispatch to response time, not the other way. We therefore believe it’s more accurate to refer to this as a mediating factor, not a confounder, and not accounting for such delay will not lead to bias.

A preliminary test indicates that a 10% increase in busy probability results in 2 seconds increased turn-out time. We do not include such analysis in this paper, since we are less sure about the data quality underlying these numbers. We do not attempt to address all aspects of response time in this work, but this could certainly be future work. Nevertheless, it is an interesting point we have brought into the Measures and Strengths and limitations sections of our revised manuscript (*line #153 and #340*).

### #R2-2

*You mentioned in lines 247 and 248 that the busy ambulance in a rural area affected the response, which can be understood due to the long distances and extended response time. Hence, I would like to discuss an explanation if this is due to the type of acute medical emergency. Was it affected by some demographic variables like age?*

**Author’s reply (ref line #277 to #279 in updated manuscript)**

This is a good question, which we have tried to address as best we can by studying if there were any relationships between type of incidents and probability of busy ambulances in our supplementary analysis balance test *(line #217 and #268*), see also *S1 Table*. Since we didn’t have patient data in this study, we could not test for differences in age, sex or other patient characteristics, but this will be addressed in future work (*line #360*). See also answer to comment #R1-12 and #R1-16.

Although long distances should be the prime suspect here, the question of case mix must of course be considered. We have amended the Strengths and limitations to address this point (*line #354*).

### #R2-3

*Further, you mentioned in the method that you used machine learning technique:*

#R2-3a

*This has to be reflected in the title as it is fundamental.*

**Author’s reply**

As suggested, we have changed the title to better reflect the method (*line #1*).

#### #R2-3b

*Further, I hoped to see in the method and the discussion information about the algorithms you used and why you chose them. This would be important since you used probabilistic methods. Further, to make the study replicable, it is important to mention in the statistics how you handled the missing values and provide a deeper description of your variables and how you evaluated that the model you used is efficient in prediction and how accurate it was. These details are important and give more credibility to the results. These details should also be visible in the abstract.*

**Author’s reply**

Our method was not sufficiently described. We have updated our Measures section to bring clarity to our underlying method (*line #153*). See also answer to comment *#R1-6* for more on missing values.

#### #R2-3c

*In the limitations, I would advise adding a few sentences about the statistical bias and the limitation of deploying the model you built.*

**Author’s reply**

We agree. Please see our revised strengths and limitations section *(line #340*). See also answer to comment *#R1-14*

#### #R2-3d

*This was about prediction; in every model, there would be a true positive, true negative, false positive, and false negative, which was very important to discuss.*

**Author’s reply**

Thanks to this question, we realise that the language was misleading here, since we did not, in fact, attempt to predict which ambulances were to respond, rather which ambulances were candidates to respond. To avoid misunderstandings, we have now pointed this out, and changed the wording throughout the paper.

There is of course prediction underlying this, and the question of prediction errors is valid. However, we attempted to predict an unobserved quantity, so assessing prediction errors is tricky. On the other hand, we may reason about this, considering the way the predictions are used for analysing busy ambulances, and considering that the quality of the prediction may impact bias and precision: First, a better prediction should clearly give more precise estimates. Also, by analysing within small geographical units at comparable times, false predictions would likely not associated substantially with response time. Second, a better prediction that better pinpointed where there was a problem with busy ambulances (positive prediction value), would likely yield stronger effect estimates. In total the impact of prediction quality would be a bias towards the null. This reasoning was done when designing the analysis, but did not receive the attention it deserved in the paper. We have amended the strengths and limitations section to ameliorate this *(line #345).*

#### #R2-3e

*If you used one model, I don’t think that would be sufficient as another algorithm might perform better, which, unfortunately, you did not mention. So please, I would suggest adding a further discussion about this.*

**Author’s reply**

We hope that the extension of the discussion discussed above *(d),* addresses this point as well *(line #340).*

1. Asheim A. Ambulance_response. In: andreasasheim/Ambulance_response [Internet]. 1 Aug 2023. Available: https://github.com/andreasasheim/Ambulance_response
2. Asheim A. Ambulance_response. In: andreasasheim/Ambulance_response [Internet]. 1 Aug 2023. Available: https://github.com/andreasasheim/Ambulance_response
3. Gelman, A. (2019). Don’t calculate post-hoc power using observed estimate of effect size. Annals of surgery, 269(1), e9-e10.
4. Zhang, Y., Hedo, R., Rivera, A., Rull, R., Richardson, S., & Tu, X. M. (2019). Post hoc power analysis: is it an informative and meaningful analysis?. General psychiatry, 32(4).
5. Asheim A. Ambulance_response. In: andreasasheim/Ambulance_response [Internet]. 1 Aug 2023. Available: https://github.com/andreasasheim/Ambulance_response
